# Killing them softly: managing pathogen polymorphism and virulence in spatially variable environments

**DOI:** 10.1016/j.pt.2013.07.002

**Published:** 2013-09

**Authors:** Pedro F. Vale

**Affiliations:** Centre for Immunity, Infection, and Evolution and Institute of Evolutionary Biology, School of Biological Sciences, University of Edinburgh, Ashworth Laboratories, West Mains Road, Edinburgh EH9 3JT, UK

**Keywords:** pathogen polymorphism, hard selection, soft selection, epidemics, virulence evolution, co-infection

## Abstract

Understanding why pathogen populations are genetically variable is vital because genetic variation fuels evolution, which often hampers disease control efforts. Here I argue that classical models of evolution in spatially variable environments – specifically, models of hard and soft selection – provide a useful framework to understand the maintenance of pathogen polymorphism and the evolution of virulence. First, the similarities between models of hard and soft selection and pathogen life cycles are described, highlighting how the type and timing of pathogen control measures impose density regulation that may affect both the level of pathogen polymorphism and virulence. The article concludes with an outline of potential lines of future theoretical and experimental work.

## Highlights

• Understanding the maintenance of pathogen variation is central to disease control.
• Classical metapopulation models make useful predictions about polymorphism.
• The timing of pathogen regulation is key to pathogen polymorphism and virulence.

## Pathogen polymorphism in spatially heterogeneous environments

The ability of pathogens to infect and cause harm to their hosts varies widely [1]. Much of this observed phenotypic variation is underlined by genetic variation [1–5], and therefore directly affects pathogen fitness and their potential response to selection [6,7]. Thus, understanding the conditions that maintain pathogen polymorphism would aid our ability to manage the risk of disease spread, disease evolution, and host shifts [1,4,8]. What, then, maintains genetic variation in pathogen populations? Understanding the maintenance of polymorphism in natural populations is a long-standing focus of evolutionary biology [9,10]. The interest arises because genetic variation would be expected to decrease as selection favours alleles that result in higher fitness, but this is contradicted by the observation of widespread genetic variation in traits affecting fitness [11,12]. One key point is that environments vary both in space and time, and because different alleles are advantageous in different environments, when populations become adapted to local environmental conditions, the result is the maintenance of genetic polymorphism across the whole metapopulation [13–16].

The environment faced by pathogens is never homogeneous, and both the genetic identity of the hosts encountered and the abiotic environment are often variable [7,17,18], with known implications for the epidemiology [19–22] and evolution of disease [7,18,23]. Hosts are a particularly important environment to which pathogens must adapt [24,25], and because pathogen genotypes will grow better in some host genotypes and worse in others, environment-dependent pathogen fitness may therefore lead to locally adapted pathogen genotypes and maintain pathogen polymorphism [16,23,26]. Testing for host- or environment-specific pathogen fitness [27–29] is therefore an important first step towards understanding whether pathogen polymorphism is likely to be maintained.

Beyond differences in pathogen fitness across environments, it is important to account for the spatial nature of pathogen life cycles. Following a period of within-host growth, pathogens must transmit, and the success or failure of transmitting to a new host will not only depend on the number of transmission stages produced in the current host but also on what happens between hosts. For pathogens with passive or air-borne horizontal transmission, transmission stages originating from several hosts may disperse together and mix, generating competition between pathogen genotypes for the colonization of novel hosts. This adds an additional level of selection (in addition to within-host selection) that may be severe if suitable hosts are scarce. Furthermore, pathogen densities may be regulated at any of these stages. For example, different types of antimicrobial or anti-parasitic measures may be applied at different stages of pathogen life cycles to target pathogen numbers by controlling within-host growth, reducing the transmission stages produced, or blocking infection in novel susceptible hosts [30]. Here I argue that accounting for the timing of each of these events, as well as the type of density regulation, may inform on whether pathogen polymorphism is more likely to be maintained or reduced.

## Hard and soft selection and pathogen polymorphism

The spatially structured nature of pathogen life cycles shares many features of classical models of evolution in spatially variable environments (Box 1). Although these models are well known to population geneticists, they are less common in the parasitological literature. However, as described in Box 1, the maintenance of polymorphism depends critically on when and how population densities are regulated. Interpreting pathogen life cycles in light of models of hard and soft selection (see Glossary) may therefore be relevant to our understanding of pathogen life cycles and also lend new insight into the maintenance of pathogen polymorphism. For example, treatments that regulate pathogen densities before transmission between hosts are beneficial locally by reducing disease prevalence, but if regulation results in frequency- and density-dependent selection (soft selection), genetic variation is predicted to be maintained under various conditions (Box 1). Alternatively, regulation applied after pathogens have dispersed from infected hosts and mixed with the total pool of infectious stages would result in density- and frequency-independent selection (hard selection), which is predicted to maintain less polymorphism. Therefore, both the timing and the specificity of treatment is important – applying control locally is more likely to impose soft selection, whereas more broad spectrum prophylactic treatments are more likely to lead to hard selection. It is worth noting, however, that local regulation might also lead to hard selection under some scenarios. For example, if treatment is not applied equally across all hosts, local regulation will result in variable numbers of dispersing parasites from each host or patch. In this case hard selection will occur if parasites disperse randomly to new hosts, but soft selection will occur if dispersing individuals are able to choose the best possible habitat (see model 3 in [31]), which might be the case for some parasites [32,33].

Applying the framework of hard and soft selection to pathogens is therefore especially useful when infection spreads through a host population that is clearly structured in space. For example, the 2001 foot-and-mouth disease (FMD) outbreak in the UK was greatly influenced by migration of animals between farms and by their mixing in livestock markets [34,35]. Restricting all movement between farms resulted in an appreciable drop in transmission [35] but was not a viable long-term strategy. Control measures focused instead on vaccination of infected farms, but vaccine-escape variants of the FMD virus quickly evolved [36,37]. A general culling of infected livestock, at enormous loss, ultimately controlled the epidemic. The framework of hard and soft selection would predict that imposing hard selection on the FMD virus, perhaps by using mass drug administration in cattle farms surrounding the infection foci (allowing the pathogen to disperse and mix before regulation), would be more likely to reduce viral polymorphism and perhaps delay the evolution of vaccine-escape mutants. Although this prediction would depend on several variables (e.g., the size of the farms and the rate of cattle migration), modelling the evolution of FMD within a framework of hard and soft selection tailored its biological details would be potentially useful.

Another potential application of models of hard and soft selection is pest control (which, incidentally, was the original inspiration for Levins’ metapopulation model of evolution in subdivided populations [38]). A pressing question in pest control is how best to apply pesticides to achieve eradication while also avoiding the evolution of resistance. In practice, managing resistance is complicated mainly by a lack of knowledge regarding the relative rates of gene flow between pest sub-populations, the ideal size of the treatment area, and the costs associated with pesticide resistance [39,40]. The framework of hard and soft selection could be useful to manage the evolution of pathogenic fungal disease when faced with spatial variation in pesticide use [38,41]. Local pesticide use may reduce the density of disease in the patches where it is applied, but by regulating pest populations locally before mixing, it also potentially imposes soft selection, which could maintain polymorphism. Instead, identifying a source of infection and treating neighbouring patches is more likely to result in hard selection (and reduce fungal polymorphism over time) because regulation of the fungal pathogen density would only occur after within-patch selection and pathogen mixing. Reducing polymorphism could slow the evolution of pesticide resistance, extending the amount of time for which pesticides are effective.

We may even go beyond genetic variation within single-species infections and consider co-infection by multiple species. Most human and animal species suffer co-infection by multiple pathogen and parasite species [42–44], and interactions between co-infecting pathogens are known to have important consequences for the spread and severity of disease [43–46]. Understanding whether control measures are more likely to maintain co-infecting pathogens or to favour one of them is therefore important. The same principles of hard and soft selection apply with multiple species. Targeting the most prevalent pathogen locally imposes density-dependent regulation and is more likely to maintain a variable (multiple) infection compared to broad-spectrum (density-independent) control measures applied after pathogens have had a chance to mix and disperse.

## Hard and soft selection and the evolution of virulence

Beyond the potential effects on the maintenance of polymorphism, imposing hard or soft selection can also influence the evolution of virulence. Chao and colleagues connected these concepts to kin selection models of virulence evolution [47]. They considered a simple co-infection scenario in which two pathogen types compete for limited host resources. Kin selection theory predicts that the genetic relatedness of the two co-infecting pathogen types will affect the outcome of virulence evolution (reviewed in [48]); highly related co-infecting pathogens are more likely to evolve altruistic behaviours such as competitive restraint, whereas low relatedness is more likely to result in strong within-host competition, favoring faster growing strains and potentially more severe infections [48].

**Figure 1:**
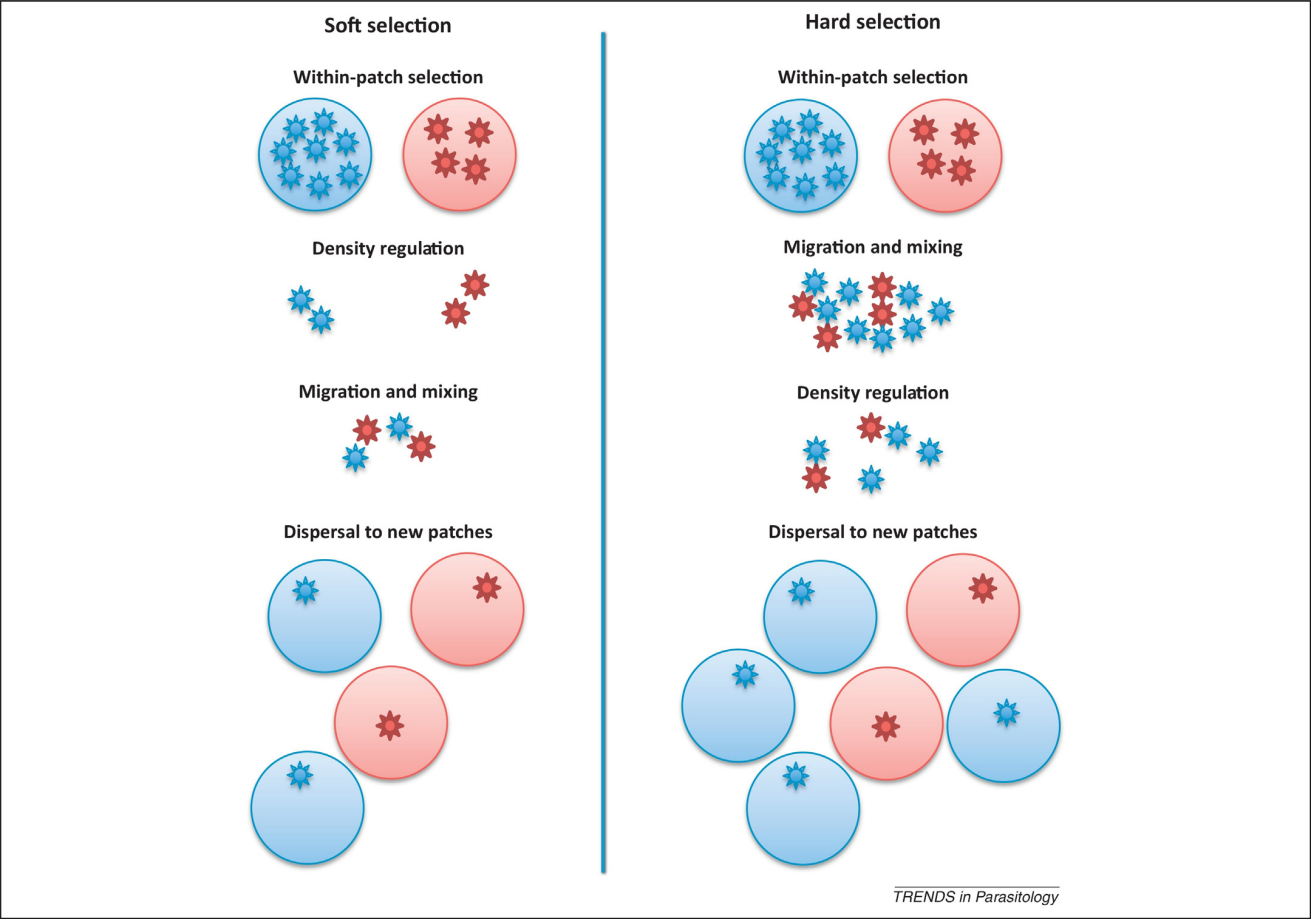
A diagram of soft and hard selection. Under soft selection, density regulation occurs before individuals disperse and mix. Under hard selection, genotypes leave their patches and mix, and regulation is applied to the whole population independently of the frequency of each genotype.

In turn, relatedness will be highly dependent on whether pathogen populations experience hard or soft selection. Under hard selection, density- and frequency-independent selection will favor pathogen types that are prevalent globally (Figure 1). This type of regulation therefore selects for pathogens with high growth rates, which may also be associated with increased virulence [49–51]. By contrast, under soft selection pathogen densities are regulated before they disperse from their hosts, which results in frequency- and density-dependent selection (Figure 1), so the probability that a new host will be co-infected at each transmission event is relatively high. Pathogen relatedness within an infected host is therefore more likely to be relatively low under soft selection, and the resulting conflict between unrelated strains could lead competing pathogens either to overexploit their hosts or mutually inhibit within-host growth and virulence [45]. We may therefore be more likely to observe the evolution of strategies that result in reduced virulence under soft selection compared to hard selection [52]. Imposing pathogen control measures at different stages of an infection cycle could therefore also affect how virulence evolves [53,54]. However, to my knowledge there are currently no direct tests of virulence evolution by imposing treatments of hard and soft selection.

## Concluding remarks and future perspectives

Herein, the parallels between pathogen life cycles and classic models of selection in subdivided populations have been illustrated under the premise that managing pathogens should rely on a full understanding of hard and soft selection processes. Throughout this article I have argued that it might be desirable to reduce genetic variation in pathogen populations, but this could be risky if the resulting fittest genotype is also resistant to pathogen control measures. Although soft selection may help to maintain variation, it does so by allowing less-adapted genotypes to coexist with fitter ones, and it therefore helps to maintain a certain level of maladaptation that, if well managed, can be useful from the perspective of disease control. In other contexts, when microbial agents are used as bio-control against pests, it may instead be preferable to maintain a large amount of standing genetic variation in order to avoid the evolution of resistance to the bio-control [55]. Models of hard and soft selection are therefore useful tools for understanding pathogen life cycles and for making predictions about polymorphism and virulence evolution, but the choice of which regime to impose is likely to depend on the parasite life cycle and the specific context (for example, whether drug resistance is already frequent). Below, some important points are discussed that should be addressed in future research (Box 2).

### More experimental tests of theory

Despite numerous theoretical studies on models of hard and soft selection, there is surprisingly little empirical work testing their predictions. Even the most consistent prediction arising from theory, that hard selection maintains less polymorphism than soft selection, has yet to be clearly demonstrated. Bell [56] presented one of the few attempts to test these predictions experimentally by imposing different forms of regulation on a mixture of *Chlamydomonas* strains kept in a heterogeneous environment for 50 generations. Regardless of the type of density regulation, substantial levels of genetic variance were maintained. This unexpected result may perhaps be explained by the duration of the selection treatment or the specific nature of the environmental heterogeneity that was imposed, which was comprised of a mixture of nutrients and not the classically defined spatially discrete patches [14]. So clearly there is a need for more experiments testing these predictions under different types of environmental variation. Again, it would be particularly useful to test these predictions in the context of pathogen transmission. For example, controlled infections in host–pathogen model systems would allow serial passage of a variable population of pathogens on their hosts, and the level of pathogen variation under hard and soft selection could be monitored.

### Measuring relevant polymorphism

Although it is possible to quantify genetic variation in a number of different pathogen traits, it is important to recognize that models of hard and soft selection make predictions about the variance in fitness that each form of regulation may maintain [57–59]. Naturally, it is possible that measuring variance in a trait that does not correlate well with fitness might not yield the expected prediction in terms of maintained polymorphism. The most direct way to assess the maintenance of polymorphism in fitness-related traits would be to measure the change in frequency of different pathogen strains in a mixed infection when hard or soft selection is imposed experimentally, because this is a direct outcome of differences in fitness. This makes microbial systems especially attractive for such studies owing to the available genetic toolbox that allows specific strains to be tracked and quantified [60–62]. Alternatively, when such tools are not available, measuring the phenotypic variance in fitness before and after imposing different forms of density regulation might be a good approximation of the underlying genetic variance maintained by hard and soft selection [56].

### Models that accurately describe pathogen life cycles

Predictions of how hard and soft selection affect polymorphism are particularly contingent on the underlying assumptions of the models. For example, some models in the hard/soft selection literature assume environments are symmetrical regarding their frequency and productivity, largely for the sake of simplicity (e.g., [63].) However, it is also well known that habitat frequencies and their relative carrying capacities are key to the outcome of local adaptation [39,40,64]. Another key aspect is how to impose density regulation in practice, because whatever the timing of regulation, if different hosts contribute different numbers of pathogen genotypes to the next transmission event, the result will be hard selection. However, recent theoretical work considered a model with incomplete levels of both soft and hard selection and confirmed the classical result that polymorphism is never maintained under hard selection but always has the chance of being maintained under soft selection [63].

A useful way forward would be to modify models of hard and soft selection to generate predictions for specific pathogen life cycles, in which host frequency and quality, density regulation, and the extent of dispersal may be modified in a biologically meaningful way. Much theoretical and experimental work has already shown how different levels of migration between patches may affect local adaptation [65–67], and it may be fruitful to extend these predictions to host–pathogen systems and the question of how modifying pathogen migration (i.e., transmission) may affect their evolution [67]. For example, it would be useful to account for complex life cycles in which pathogens go through several hosts or species, as knowledge about which stage to target by management is highly relevant. Models of this sort are currently lacking, but useful information can be gained by analysing the sequences of regulation, migration, and selection of such complex life cycles [31]. Models that consider that local adaptation affects the carrying capacity of the habitat (e.g., Model 3 in [31,68]) may be particularly useful, as they reflect the outcome of pathogen within-host adaptation on host fitness. For instance, in plant parasites, plant output is generally highly dependent on the level of within-host parasite adaptation [69]. Such an iterative approach between theory and experiment will be the key to the successful use of models of evolution in spatially structured populations to understand and manage pathogen polymorphism.

## Box 1 Models of hard and soft selection

The specific sequence of within-patch selection, mixing, dispersal, and density regulation has been extensively studied in models of selection in spatially structured populations. Specifically, Levene and Dempster's models of ‘hard’ and ‘soft’ selection [58,59] offer a useful framework within which to understand and potentially manage pathogen polymorphism. Soft and hard do not refer to the relative strength of selection acting on populations. Instead, the terms were borrowed from international money exchange and refer to soft and hard currencies; they were only later used to refer to the Levene and Dempster models as two alternative mechanisms of evolution in subdivided populations [57].

Both models consider a genetically variable population composed of two or more competing genotypes and inhabiting a spatially structured environment composed of two or more kinds of habitats or patches, where fitness varies across patch types. In both types of models, populations go through a life cycle with several stages of within-patch selection, migration between patches, mixing, and density regulation (Figure 1). The crucial difference between soft and hard selection is the timing and type of regulation. Under soft selection, regulation happens in the patch before migration and mixing, and results in density- and frequency-dependent selection. This type of density regulation therefore reduces the relative effects of within-patch selection by fixing the contribution of each patch, thereby maintaining otherwise less fit genotypes (Figure 1).

By contrast, under hard selection, after a period of within-patch selection individuals are allowed to migrate and mix, and only then is density regulation applied on the whole population. As a result, selection is frequency- and density-independent, and genotypes with high within-patch fitness are over-represented in the global mix and are more likely to survive and find a new patch (Figure 1). This ultimately selects for the genotypes that do best in the habitats available to them, and over time it will select against low-fitness genotypes, thereby reducing the amount of genetic variation [57]. Notably, in the absence of regulation, the result is also hard selection, favouring the genotype with highest within-patch fitness.

Models of hard and soft selection have been extensively studied theoretically (reviewed in [68]) and have been modified to change the order of each life cycle event [70,71], to include habitat choice of the population under selection [31,68], or to consider intermediate levels of both soft and hard selection [63]. The predictions resulting from this work are remarkably consistent; hard selection should rarely maintain polymorphism, which is more likely under soft selection, depending mainly on the degree of dispersal and the difference in initial fitness of the competing genotypes.

## Box 2 Outstanding questions

• What maintains pathogen polymorphism?
• Does soft selection maintain more pathogen polymorphism than hard selection?
• How will hard or soft selection affect the evolution of virulence?
• Is it feasible to impose hard or soft selection by varying the timing of pathogen control measures?
• Can we modify models of hard and soft selection to understand specific pathogen life cycles?
• What are the most appropriate systems for testing hard and soft selection experimentally?

## Glossary

Density regulation: a reduction in the number (the density) of individuals of a population.
Hard selection: the result of regulation that is frequency and density independent, usually occurring on the whole population after genotypes have migrated from their patches and mixed.
Kin selection theory: a theory of natural selection that takes into account how the relatedness of individuals within a population influences the evolution of social traits.
Metapopulation: a group of spatially separated populations of the same species.
Mixing: the coming together of individuals originating from different habitats or patches after a period of within-patch selection. Mixing may result in competition between individuals for the colonization of new patches. In the context of infection, mixing may occur when transmission-stage pathogens disperse from one host to infect another.
Relatedness: a measure of genetic similarity between individuals within a population.
Soft selection: in a spatially structured environment, the result of frequency- and density-dependent regulation occurring locally before genotypes migrate and mix.
Transmission stage: the life cycle stage of a pathogen or parasite that is transmitted between hosts.
Virulence: the reduction in host health or fitness owing to infection. In evolutionary models, virulence is often defined as host mortality, but it can be any trait that directly or indirectly affects the lifetime reproductive success (fitness) of the host.
Within-patch selection: the fitness outcome of growth in a given environment. In the context of infection, this is roughly the number of transmission stages a pathogen can produce during an infection cycle.
